# A Walk in the Park: A Case of Babesiosis in the South Bronx

**DOI:** 10.5811/cpcem.2017.8.35924

**Published:** 2018-01-11

**Authors:** Christina Hajicharalambous, Mohammad Rattu, Scott Leuchten

**Affiliations:** * Saint Barnabas Hospital Health System, Department of Emergency Medicine, Bronx, New York; † Inspira Health Network, Department of Family Medicine, Vineland, New Jersey

## Abstract

Babesiosis, mainly endemic within the Northeastern and upper Midwestern regions of the United States, is a zoonotic disease that invades and lyses red blood cells, which can result in hemolytic anemia. Its decreased incidence in comparison to Lyme disease is often attributed to the greater asymptomatic infection proportion and insufficient physician awareness or suspicion of this disease. Here we describe a case of undifferentiated febrile illness with hemolytic anemia that yielded the diagnosis of babesiosis.

## INTRODUCTION

Babesiosis is endemic within the Northeastern and upper Midwestern regions of the United States and is a reportable disease in 27 states.^1^ *Babesia microti* is the predominant species and mimics the progression of Lyme disease due to having the same vector, the black-legged tick *Ixodes scapularis*. According to national reported disease cases, the infection prevalence rate of *Borrelia burgdorferi* is approximately 25 times greater than *B. microti*.^2^ This discrepancy in rates is twofold. Younger individuals often have minimal symptoms and do not seek medical attention. In a cohort study by Krause et al.,^3^ asymptomatic *B. microti* infections were found in about 20% of adults and half of children in endemic Rhode Island. The lower reported incidence is also attributed to difficulty in diagnosis, lack of physician awareness and deficiency of concomitant testing for babesiosis when suspicious for Lyme disease.^2^

The parasite is spread from the young nymph stage, seeking blood during warm months. Humans are incidentally bitten by infected ticks during a blood meal, from which the tick will introduce sporozoites into the human host. Sporozoites will then reach the blood and invade erythrocytes and differentiate into trophozoites. Lysis of the infected red blood cells leads to the most common clinical manifestation of the disease, hemolytic anemia. Fragmented red blood cells can further cause renal failure. T cells that express the cluster of differentiation, four surface protein (CD4-positive T cells), are needed for adequate response and resolution of *B. microti* infection according to studies done on mice.^4^ As a result, the immunocompromised population, including those with human immunodeficiency virus, malignancy, or on immunosuppressive treatments, is at higher risk for complications with mortality rates reported as high as 20%, compared to 5–9% of all hospitalized patients with the disease. Other risk factors for development of severe disease are those older than 50 years of age or patients with previous splenectomy. 1

## CASE REPORT

A 66-year-old male with past medical history significant for hypertension, insulin-dependent diabetes, hyperlipidemia, and chronic kidney disease presented to the emergency department with progressive dyspnea and generalized weakness over the prior four days. He could previously walk up three flights of stairs without difficulty, but now could only walk up one flight before becoming dyspneic. He had associated tactile fever and weakness, which he described as decreased stamina. He also noted the chronic swelling of his bilateral lower extremities worsening over the same time period. He was not pale or icteric on skin examination. He denied associated orthopnea, cough, headache, abdominal pain, urinary complaints, sick contacts or recent travel.

On initial arrival, the patient was febrile to 38.2 degrees Celsius (°C), with a blood pressure of 131/71 millimeters of mercury (HHmg), heart rate of 80 beats per minute, respiratory rate of 18 per minute and saturating 96% on room air. He was a nontoxic-appearing obese male who was able to speak in full sentences. His heart was regular in rate and rhythm but had a grade two systolic ejection murmur. His lung sounds were clear with normal respiratory effort and symmetric chest expansion and without abnormal breath sounds. His abdominal exam was unremarkable. He had plus two pitting edema bilaterally without calf tenderness and his skin was warm without skin eruptions.

His initial laboratory evaluation was remarkable for a mild anemia (hemoglobin and hematocrit [H and H] 10.3 grams per deciliter/ 29.2%), mild transaminitis (alanine aminotransferase [ALT] and aspartate aminotransferase [AST] of 56 international units per liter [IU/L] and 66 IU/L respectively) and thrombocytopenia at 88,000 per microliter (uL). The patient was admitted to the general medical floor for sepsis with unknown source and dyspnea. He was persistently febrile with a maximum temperature of up to 39.4°C despite treatment with broad spectrum antibiotics. On hospital day two, the patient’s H and H precipitously dropped from 10.3 gm/dL /29.2% on admission to 6.0 gm/dL /16.9% with a concomitant increase in blood urea nitrogen (BUN)/creatinine (Cr) and ALT/AST that reached maximum levels of 94/10.4 milligrams per deciliter and 63/78IU/L respectively, and a thrombocytopenia that fell to 66,000/uL. It was determined that the patient was hemolysing with reticulocytes of 2.7% and lactate dehydrogenase of 631 IU/L. Upon further questioning, the patient stated that three weeks prior he had been at a local state park for a day trip. He denied drinking water from any source and did not recall any tick or other insect bites.

**Image 1:**
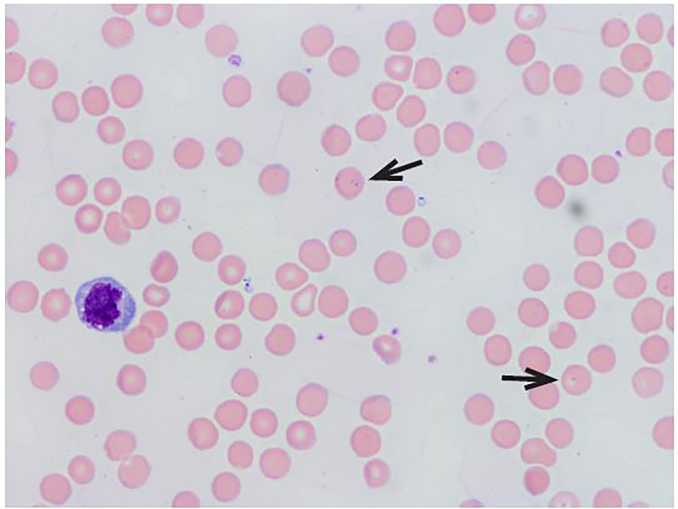
Peripheral blood smear of patient, with arrows showing intraerythrocytic parasites seen in babesiosis

Given his acute decompensation and hemolytic anemia, he was transferred to an intensive care unit step-down unit for close monitoring. Blood, urine and sputum cultures were all negative, and a repeat radiograph of the chest was also unremarkable. A peripheral blood smear (Image 1) was performed with the given clinical picture of fever, thrombocytopenia and hemolytic anemia, and the diagnosis of babesiosis was ultimately made. Atovaquone was added to the patient’s medication regimen with improvement in both clinical status and renal and hepatic function. On hospital day seven, he was discharged home in stable condition without sequelae from illness.

CPC-EM Capsule

What do we already know about this clinical entity?

Babesiosis is a parasitic disease that presents similarly to Lyme disease with fever, anemia and thrombocytopenia. It can cause severe illness in immunocompromised patients.

What makes this presentation of disease reportable?

Babesiosis is a reportable disease as it is endemic to the northeast and upper midwest regions of the United States.

What is the major learning point?

The threshold to ordering a peripheral smear should be low and considered as a concomitant test with Lyme titers.

How might this improve emergency medicine practice?

Diagnosis in the emergency department can facilitate initiation of correct treatment in hope to hasten the complications of severe parasite load.

## DISCUSSION

The most frequently reported presenting symptoms of babesiosis are fever (91%), fatigue, malaise, weakness (91%), and shaking chills (77%). When adding hemolytic anemia, this presentation still holds a broad differential including malaria, babesiosis, ehrlichiosis, relapsing fever, Chagas disease, thrombotic thrombocytopenic purpura, immune thrombocytopenic purpura, hemolytic uremic syndrome, and disseminated intravascular coagulation among other causes. This patient initially presented with vague symptoms associated with babesiosis, but did not show classic laboratory findings of disease until hospital day two. Early in the presentation, patients with babesiosis may have nonspecific findings similar to our patient. A peripheral smear performed early in the disease process may not be conclusive for babesiosis. For this reason, knowledge of babesiosis is important for emergency physicians in order to recognize this condition and prevent the downstream complications.

More severe complications from babesiosis include congestive heart failure, disseminated intravascular coagulation, myocardial infarction, renal failure and acute respiratory distress syndrome.^6^ These are more commonly seen in the elderly and the immunocompromised patients and are associated with higher parasite loads.

**Image 2:**
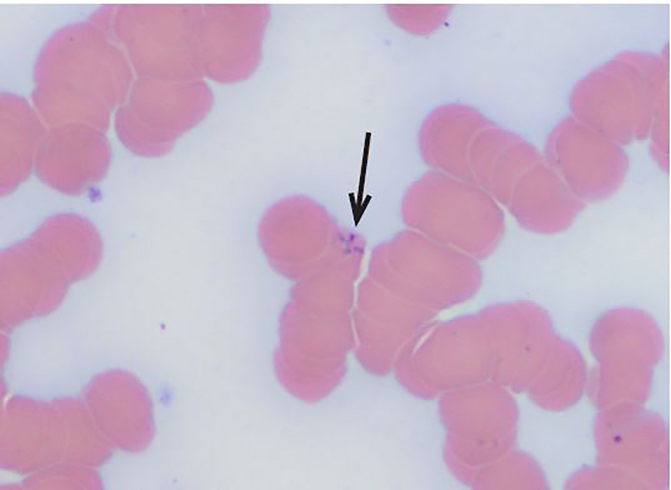
Patient’s blood smear with arrow showing intracellular formation of tetrads classically seen in babesiosis. This is also commonly described as a Maltese cross.

The diagnosis of babesiosis can be made by Giemsa or Wright staining of peripheral smear through visualization of intraerythrocytic parasites. A distinct feature of the parasite is the formation of tetrads (Image 2) giving the characteristic appearance of a Maltese cross. Polymerase chain reaction (PCR) can also assist in the diagnosis and is indicated when clinical suspicion is high and multiple peripheral smears do not give the diagnosis. PCR has also been shown to be more sensitive and comparatively specific in the acute diagnosis of the disease.^5^

For the symptomatic patient, symptoms may develop as late as one to four weeks post exposure. Thus, it is important for emergency physicians to ask about recent travel, including in the prior four to six weeks, when obtaining a history. Treatment recommendations for moderate disease, as per the Centers for Disease Control and Prevention, include at least 7–10 days of two prescription medications, atovaquone plus azithromycin orally, or clindamycin plus quinine.^7^ Studies have shown that the treatment with the latter had increased rate of adverse effects, 15% vs. 72% respectively, with tinnitus being the most common.^7^ It did, however, show better promise in the treatment outcome for severely immunocompromised and decompensating patients.^8^ In immunosuppressed patients, treatment includes high-dose azithromycin plus atovaquone for six weeks and renal exchange transfusions if parasite load exceeds 10%.

## CONCLUSION

The diagnosis of babesiosis is difficult to make in the ED particularly when the clinical presentation is early in the course of the disease and suspicion is low, even in endemic areas. Keeping babesiosis in the differential when patients present with fever and anemia with thrombocytopenia is important, and a peripheral smear should be performed if suspicion exists. The threshold for ordering a peripheral smear should be low and considered as a concomitant test with Lyme titers. Although an urban setting is not typically associated with babesiosis, obtaining a travel history is critical and may serve as supporting evidence of infectious etiology. Even when suspicion is high and a peripheral smear is negative, PCR can be considered as it is a more sensitive method of detection.
